# RETRACTION: Survival and stability of free and encapsulated probiotic bacteria under simulated gastrointestinal conditions and in ice cream

**DOI:** 10.1002/fsn3.4094

**Published:** 2024-04-05

## Abstract

**RETRACTION:** Muhammad Afzaal, Azmat Ullah Khan, Farhan Saeed, Muhammad Sajid Arshad, Muhammad Asif Khan, Muhammad Saeed, Abid Aslam Maan, Muhammad Kashif Khan, Zoria Ismail, Aftab Ahmed, Tabussam Tufail, Huda Ateeq, and Faqir Muhammad Anjum, “Survival and stability of free and encapsulated probiotic bacteria under simulated gastrointestinal conditions and in ice cream,” *Food Science & Nutrition* 8 (2020): 1649–1656, https://doi.org/10.1002/fsn3.1451.

The above article, published online on 17 February 2020 in Wiley Online Library (wileyonlinelibrary.com), has been retracted by agreement between the journal's Editor‐in‐Chief, Y. Martin Lo, and Wiley Periodicals, LLC. The retraction has been agreed to following concerns about the peer review process. The editorial office found unambiguous evidence that the manuscript was accepted solely based on compromised and insufficient reviewer reports. This evidence was further investigated and verified by the publisher and the Editor‐in‐Chief. As a result, the conclusions of this article are not considered reliable. The authors were notified of the retraction and disagreed with this decision.

**RETRACTION:** Muhammad Afzaal, Azmat Ullah Khan, Farhan Saeed, Muhammad Sajid Arshad, Muhammad Asif Khan, Muhammad Saeed, Abid Aslam Maan, Muhammad Kashif Khan, Zoria Ismail, Aftab Ahmed, Tabussam Tufail, Huda Ateeq, and Faqir Muhammad Anjum, “Survival and stability of free and encapsulated probiotic bacteria under simulated gastrointestinal conditions and in ice cream,” *Food Science & Nutrition* 8 (2020): 1649–1656, https://doi.org/10.1002/fsn3.1451.

The above article, published online on 17 February 2020 in Wiley Online Library (wileyonlinelibrary.com), has been retracted by agreement between the journal's Editor‐in‐Chief, Y. Martin Lo, and Wiley Periodicals, LLC. The retraction has been agreed to following concerns about the peer review process. The editorial office found unambiguous evidence that the manuscript was accepted solely based on compromised and insufficient reviewer reports. This evidence was further investigated and verified by the publisher and the Editor‐in‐Chief. As a result, the conclusions of this article are not considered reliable. The authors were notified of the retraction and disagreed with this decision.

